# Repair of single-strand breaks during the cell cycle - saturation of repair capacity

**DOI:** 10.1080/19491034.2026.2673717

**Published:** 2026-05-17

**Authors:** Oskar Szelest, Agnieszka Hoang-Bujnowicz, Jurek W. Dobrucki, Mirosław Zarębski

**Affiliations:** Department of Cell Biophysics, Faculty of Biochemistry, Biophysics and Biotechnology, Jagiellonian University, Kraków, Poland

**Keywords:** DNA single-strand break, base excision repair, XRCC1, PCNA, laser microirradiation, NAD^+^

## Abstract

Imaging of recruitment of XRCC1 and PCNA to a predetermined number of DNA single-strand breaks (SSB) demonstrates that (i) short-patch base excision repair is active throughout the whole cell cycle and repairs SSBs in over 60% of nonreplicating cells, while long-patch BER is active in approximately 30% of nonreplicating cells, becomes inactive in the early S phase and shows activity again in the mid and late S phase (in 8 and 16% of cells, respectively), (ii) cells retain the capacity to respond to damage if the total number of SSBs induced within seconds does not exceed approximately 110, more breaks evoke no XRCC1 recruitment; (iii) within less than a second, a cell commits a part of the currently available pool of XRCC1 molecules to accumulation at the damage site. We hypothesize that a limit on the number of single-strand breaks that elicit the BER response can arise from limited poly-ADP-ribosylation.

## Introduction

A human cell must continuously repair large numbers of single-strand breaks (SSBs) induced by endogenous and exogenous factors. It is believed that 2 to 10,000 spontaneous depurination acts occur daily in a human cell [1]. These numerous abasic sites are filled by base excision repair (BER) process in which transient SSBs are generated. Endogenous DNA strand breaks also arise as an intermediate step during repair of oxidized bases; or because of malfunctioning of topoisomerase I. Exogenous factors that induce SSBs include ionizing radiation, ultraviolet (UV) radiation, heat shock, and various chemicals. SSBs can cause replication stress, transcriptional arrest (reviewed recently by [2]), and mutations. SSBs are efficiently repaired by single-strand break repair (SSBR), a pathway that shares late steps with base excision repair (BER). SSB repair can proceed via short-patch or long-patch BER (SP BER or LP BER).

SSBs are detected by poly [ADP-ribose] polymerases 1 and 2 (PARP1 and PARP2). By consuming NAD^+^, these enzymes transfer ADP-ribose subunits onto themselves, histones, and other proteins. Chains of ADP-ribose are formed rapidly and act as binding sites for a scaffold protein X-ray cross-complementing protein 1 (XRCC1) [3], a key factor in the SSBR pathway. XRCC1 accumulates at the damage site in high numbers [4] and forms distinct foci detectable by microscopy in less than a minute [5]. Poly-ADP-ribose (PAR) chains are rapidly degraded. The accumulated XRCC1 recruits other SSBR enzymes and is likely to remain at the damage site at least for the duration of the repair process. Further steps of the repair process can follow the short-patch or long-patch BER/SSBR pathway. In SP BER, DNA polymerase β removes the 5’-deoxyribose phosphate group and inserts the missing nucleotide. The final step involves ligation by ligase I or III. In LP BER, DNA polymerase δ or ε synthesize a 2–10 nucleotide segment, displacing the damaged DNA fragment and forming a 5’ flap. This flap is then removed by flap endonuclease 1 (FEN1), and the DNA ends are ligated by ligase I or ligase III. These steps involve proliferating cell nuclear antigen (PCNA), a component of LP BER that can interact directly with several LP BER factors, including FEN1 and DNA ligase I (reviewed by [6] and [7]).

PCNA is involved not only in LP BER, but also in several other repair pathways as well [8–11]. However, its primary role is in canonical DNA replication. The PCNA homotrimer encircles DNA and serves as a processivity factor for polymerases. Thus, when SSBs are induced in the S phase, PCNA can be expected to be involved in DNA replication and DNA repair. This raises the question as to whether the involvement of PCNA in both processes decreases the availability of this protein and slows their rates.

While cells repair endogenous damage efficiently, the question arises regarding saturation of the repair capacity, that is, the cellular capacity to handle additional, often extensive damage induced by exogenous factors. Studies of in vitro cultured rat brain tumor cells demonstrated that, as the dose of ionizing radiation increased, the saturation of repair processes of single- and double-strand breaks occurred [12]. Exposure to an oxygen ion beam revealed that fewer 53BP1 and RAD51 proteins were recruited to a second group of double-strand breaks than to the first one [13]. Saturation of repair capacity was also observed after exposure of cells to UV (254 nm) [14]. In *E. coli*, high doses of deoxyribosyl-dihydropyrimido[4,5-c][1,2]oxazin-7-one saturated mismatch repair [15]. Therefore, while the fact that the capacity to repair DNA lesions is limited was expected and is now well documented, the actual number of lesions that saturate this capacity and the stage of the repair pathway that acts as a limiting factor are not known. We developed a method to induce a controllable number of SSBs in individual cells and employed this approach to establish whether simultaneous involvement of PCNA in LP BER and replication influences the contribution of LP BER in SSB repair and to estimate the number of SSBs that saturate the repair capacity of a cell and result in leaving some lesions unattended.

## Materials and methods

### Cell cultures

HeLa cells were cultured in DMEM (Sigma-Aldrich, D5523-50 L) supplemented with 10% FBS (Sigma-Aldrich, F9665), 50 U/ml penicillin and 50 μg/ml streptomycin (Sigma-Aldrich, P4333), at 37°C in a humidified atmosphere (95% humidity, 5% CO_2_). Cultures were propagated in T-25 flasks (SPL Life Sciences) or 12-well polystyrene plates (Nest Biotechnology or SPL Life Sciences). Cells were subcultured every 3 to 4 days using a 0.25% trypsin solution (Sigma-Aldrich, T7409-1 G) (w/v). For confocal microscopy imaging, cells were seeded on 18 mm diameter round coverslips (thickness 0.17 mm; Menzel-Gläser, Braunschweig, Germany). The thickness of the coverslips was verified using a micrometer screw gauge. Coverslips were cleaned with 70% ethanol (POCH, Gliwice, Poland, 396 420 113) and sterilized with dry heat (160°C, 1 h).

### Immunofluorescence

For immunofluorescence staining, cells were fixed in 4% formaldehyde (diluted from a 16% stock (Electron Microscopy Sciences, 15710-S)) in PBS containing Ca^2+^ and Mg^2+^ ions for 15 min at room temperature (RT) (for anti-XRCC1 immunostaining) or in ice cold methanol (99.8%) (Sigma-Aldrich, M-3641) for 15 min at −20°C (for anti-PCNA immunostaining). Cells were permeabilized (15 min, RT) with 0.1% Triton X-100 (Sigma-Aldrich, T8787) in PBS. Blocking and antibody dilution (mouse anti-XRCC1 (Abcam, ab1838) 1:200, rabbit anti-PCNA (Abcam, ab92552) 1:200, anti-mouse FluoTag®-X2 ATTO488 (NanoTag, N2002) 1:200, anti-rabbit FluoTag®-X2 ATTO488 (NanoTag, N2402) 1:200) were performed in 3% bovine serum albumin (Sigma-Aldrich, A7906) dissolved in PBS. Incubation with primary antibodies was 1 h at RT, cells were washed with PBS and incubated with secondary antibodies for 2 h at RT.

### Transfection

Cells were transfected with the pmRFP-C1-XRCC1 plasmid (200 ng per sample) and pcDNA3.1+ mEGFP-PCNA plasmid (200 ng per sample) [16], individually or in combination. Transfections were performed with FuGENE® HD Transfection Reagent (Promega, E2311) (3,5 µl per 1000 ng of plasmid DNA) dissolved in Opti-MEM (ThermoFisher Scientific, #31985047) (50 µl mixture of medium, plasmid DNA and transfection reagent per 1000 ng plasmid DNA) when the cultures were in the logarithmic phase of growth (40–60% confluence), on coverslips placed in 12-well plates, submerged in Opti-MEM supplemented with 10% FBS (Sigma-Aldrich, F9665).

### Induction of SSBs by microirradiation

Single-strand breaks were induced using modifications of the method described previously [17]. Briefly, in studies of the dynamics of recruitment of XRCC1 or PCNA to SSBs a small subnuclear region was illuminated with a scanning beam of 488 nm laser light (120 µW measured in front of the objective lens) focused by HCX PL APO CS 63.0× NA1.40 oil objective. To induce damage, the 2.5 µm × 2.5 µm region was scanned 6 times, with 512 lines and a scan frequency of 200 Hz.

In experiments aimed at studying saturation of the repair processes, 5–30 nonoverlapping sites per cell nucleus were exposed to a stationary beam of a 488 nm laser. For each illumination point, the dose of 120 µJ of energy was delivered within 1 s (120 µW). The spots were illuminated immediately one after another.

To estimate the number of SSBs induced in the illuminated spot, we studied the probability of detecting XRCC1 recruitment as a function of energy delivered by a laser beam. The dose was adjusted by maintaining the constant light intensity of 120 µW and adjusting the time of exposure of the focused 488 nm light. The energy dose below 4 µJ did not result in the recruitment of XRCC1 in the sample of 10 cells. The dose of 4,5 µJ induced the recruitment of XRCC1 in approximately 1 in 10 cells, and the dose of 25 µJ in approximately 9 in 10 cells. Assuming that the number of induced SSBs is proportional to the energy delivered, we concluded that the dose of 120 µJ, used in studies of saturation of DNA damage described here, resulted in the induction of 11 ± 3 SSBs per illuminated spot (see also Suppl. Figure S1).

In experiments involving cells expressing mEGFP-PCNA, only cells displaying typical morphology and subnuclear localization of eGFP-PCNA (characteristic for nonreplicating cells and S phase replication (as in Suppl. Figure S2) were selected for induction of local DNA damage and further imaging.

### Confocal imaging

Cells expressing mRFP and eGFP-tagged proteins were imaged using a Leica TCS SP5 confocal microscope (Leica Microsystems, Wetzlar, Germany) equipped with the oil immersion objective HCX PL APO CS 63.0× NA1.40. Excitation wavelengths and detection bands were 488 nm and 500–550 nm for eGFP, and 594 nm and 610–700 nm for mRFP, respectively. The fluorescence of ATTO488 labeled antibodies was excited at 488 nm and detected in the 500–600 nm band.

The intensity of excitation light emerging out of an objective lens was measured using a Vega Laser & Energy Meter (Ophir Photonics, Vega, Jerusalem, Israel) equipped with PD300 300 mW Silicone Photodiode Sensor (Ophir Photonics). During time-lapse recordings, the excitation light intensities were 10 µW and 2 µW for 488 nm and 594 nm lines, respectively. PMT gain was 900 to 1000 V; offset: −0.1%. 512 × 512 pixels images were collected (pixel size 60.2 nm).

For live cell imaging, coverslips with cells were mounted in a custom-made steel holder. During imaging, cells were maintained at 37°C and in DMEM/F12 (Sigma-Aldrich, D2906) culture medium without phenol red, containing sodium bicarbonate and HEPES to maintain physiological pH (6.8–7.4).

### Image processing

The levels of fluorescence of fusion proteins varied between cells. Thus, measurements of fluorescence intensity of repair factors accumulated in the regions of active repair were calculated in relation to the fluorescence intensity of the mobile pool in the surrounding nucleoplasm. Fluorescence intensity measurements were performed on raw microscopy images using Fiji (ImageJ) [18]. Fluorescence intensity profiles (across the XRCC1 and PCNA foci) were prepared by averaging 25 image lines (equal to 1.5 μm) running through the nuclear region.

The contrast of the images shown in the paper was adjusted to make the repair foci stand out in print.

In repair saturation experiments, the mean fluorescence intensity measurement in the damaged spot was performed in a circular area with a diameter of 1.5 µm and the center located at the site of damage induction. This area of measurement encompassed the entire signal of the accumulated XRCC1 and PCNA. The mean fluorescence intensity of the signal from the mobile pool of the repair protein was measured in the circular region of the nucleus in the area at a distance from the damaged spots. The selected areas did not contain nucleoli or subnuclear XRCC1 bodies outside of the damaged region.

## Results and discussion

In order to establish the contributions of short-patch BER and long-patch BER to repair of SSBs we induced DNA breaks in cells in various stages of the cell cycle and imaged recruitment of XRCC1 (which is involved in SP BER and LP BER) and PCNA (which is involved in LP BER only) to damage sites. Subsequently, to measure the number of SSBs that saturate the DNA damage response, we induced increasing numbers of SSBs and imaged the accumulation of XRCC1. The accumulation of XRCC1 at the damage sites was regarded an indicator of the presence of cellular response to damage.

### XRCC1 recruitment and dissociation from SSBs

**Figure 1.**
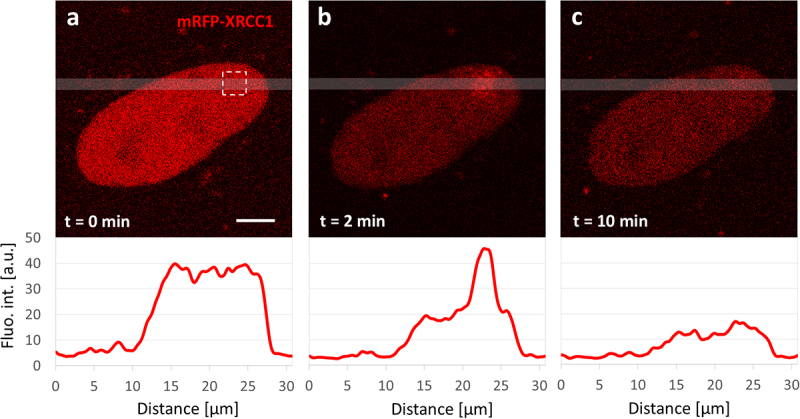
A typical example of recruitment of mRFP-XRCC1 to SSBs induced by a beam of 488 nm light in a 2.5 µm × 2.5 µm region of the cell nucleus (marked with a dashed square in (a)). (a) Image of mRFP-XRCC1 in the cell nucleus before damage induction, (b, c) 2 and 10 min after damage induction. The recruitment of mRFP-XRCC1 is followed by dissociation upon completion of repair (c). Fluorescence intensity profiles running along the transparent gray lines are shown below the images. The concentration of recruited protein is usually approximately 2 times higher in the damaged region than in the surrounding nucleoplasm. When the number of induced SSBs was higher than in this experiment, the fluorescence intensity of the accumulated mRFP-XRCC1 was correspondingly higher (see also [5]). Scale bar 5 µm. An example of mRFP-XRCC1 recruitment to SSBs induced by a stationary laser beam focused in a selected point in the cell nucleus is shown in Suppl. Figure S3 (for technical details of microirradiation, see Materials and Methods). Since XRCC1 is dynamic, a significant loss of fluorescence occurs during exposure to light inducing DNA damage.

We first analysed the cellular response to the induction of new SSBs in cells expressing mRFP-XRCC1 and eGFP-PCNA. Groups of 2–3 SSBs were induced within 2.5 µm × 2.5 µm regions (in the XY plane) in the nuclei, in cells in a logarithmically growing culture. Within the first minute, recruitment of XRCC1 to the damaged region occurred in all of these cells (Figure 1). This rapid recruitment of XRCC1 to damage confirmed that induction of 2–3 SSBs was sufficient to evoke a detectable damage response in cells of all stages of the division cycle. It is important to note that to avoid the potential influence of synchronization procedures on cellular physiology [19,20], these cells were not synchronized. The position of a cell in the cell cycle stage was established based on the pattern of eGFP-PCNA in the cell nuclei (Suppl. Figure S2).

**Figure 2.**
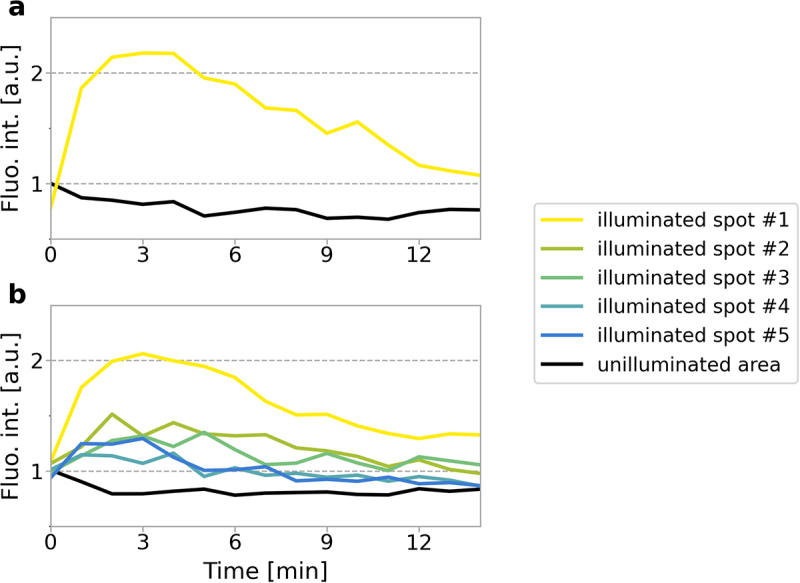
Dynamics of the recruitment of XRCC1 to SSBs induced by a beam of focused 488 nm light at one or several sites in the cell nucleus. (a) An example of changes in the fluorescence intensity of mRFP-XRCC1 accumulated at the site of induction of approx. 11 SSBs (damage induced by a stationary laser beam in one selected spot), and the accompanying level of fluorescence of the mobile pool of this protein (black), showing its decrease at the time of formation of the XRCC1 repair focus. The maximum local concentration of the accumulated XRCC1 is reached within 2–4 minutes. Dissociation begins after 2–5 minutes. Images recorded before and 3 minutes after damage induction are shown in Suppl. Figure S3. (b) An example of changes in the intensity of fluorescence of mRFP-XRCC1 that is accumulated in 5 subsequent damage sites, followed for 14 minutes after damage induction. Here, the damage was induced by a laser beam which was stopped for 1 second at five selected sites, one after another. Therefore, the recruitment of XRCC1 to the next damage site occurred while the recruitment to the previous site was still ongoing. The amount of XRCC1 that was recruited to each subsequent damage site (at a maximum, approximately 4 minutes after damage induction) was always lower than in the previous site (see Figure 5, which shows the results of similar experiments in which damage was induced in 5, 10 and 30 sites).

When DNA damage was induced in a 2.5 µm × 2.5 µm region the recruitment of a fluorescently labeled XRCC1 was characterized by the immediate formation of a diffuse cloud-like spot at the damage site (Figure 1), followed by the transition to discrete repair foci, as previously described [5]. Within 10 minutes after DNA damage induction dissociation of XRCC1, which we interpret as the completion of repair, occurred in up to 50% of cells (Suppl. Figure S4). In experiments, where SSBs were induced by a stationary laser beam focused in a selected site within the nucleus (as in Suppl. Figure S3), dissociation of XRCC1 was observed as early as 3 minutes after damage induction (Figure 2).

### PCNA recruitment to SSBs at various stages of the cell cycle

**Figure 3.**
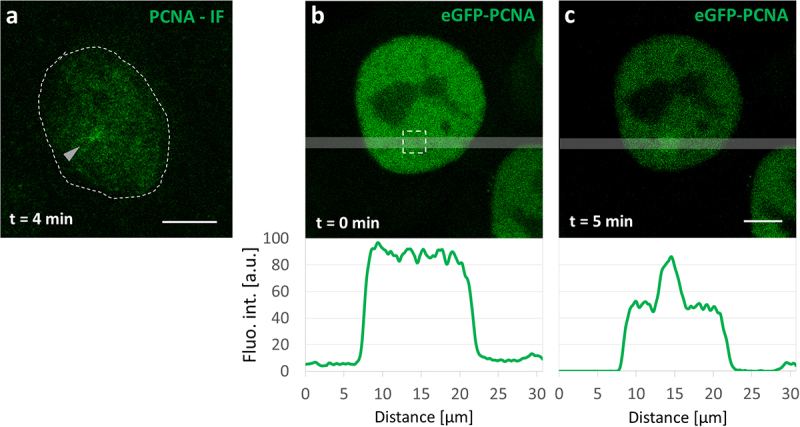
Recruitment of PCNA to SSBs imaged in a nonreplicating live cell. (a) The accumulation of PCNA at the damage site, shown by immunofluorescence (IF). The cell was fixed 4 minutes after inducing damage by a laser beam focused in the spot indicated by an arrow. (b) Image and fluorescence intensity profile running across the nucleus of a cell expressing eGFP-PCNA, along the transparent gray line, before induction of local damage (the 2.5 µm × 2.5 µm damage region is marked with a dashed square). (c) Image and fluorescence intensity profile 5 minutes after damage induction, showing recruitment of eGFP-PCNA. The general loss of fluorescence is due to photobleaching of the eGFP-PCNA pool during damage induction. Scale bars 5 µm.

**Figure 4.**
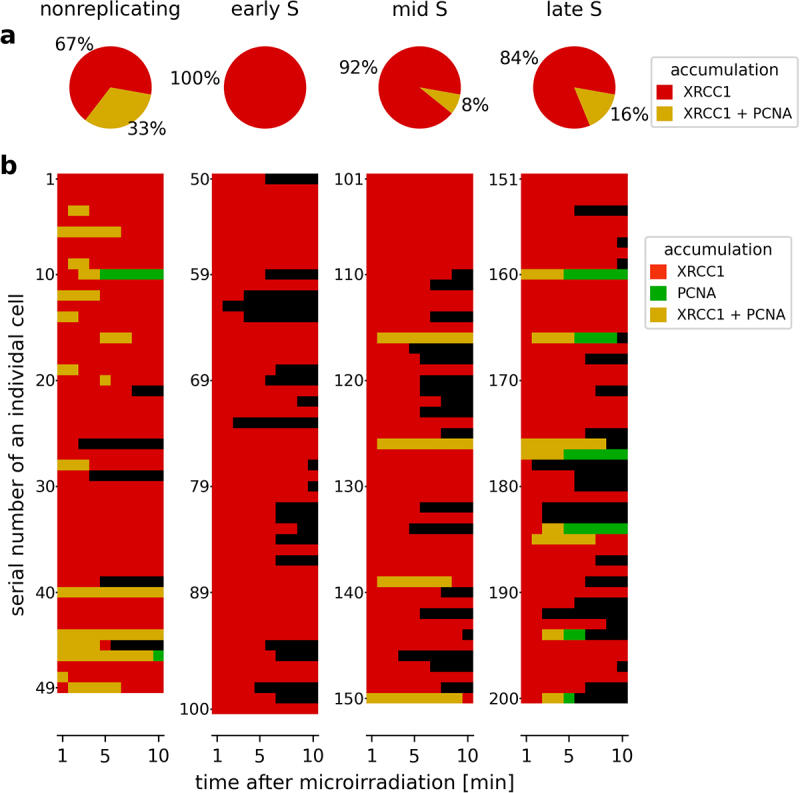
Contribution of SP BER and LP BER to total SSB repair in subsequent stages of the cell cycle. (a) Percentage of cells with recruitment of mRFP-XRCC1 and eGFP-PCNA to the same SSB sites, in subsequent stages of the cell cycle. (b) Data from panel (a), broken down into individual cells, showing recruitment of mRFP-XRCC1 and eGFP-PCNA to the same SSB site, in subsequent stages of the cell cycle (imaged for 10 min after damage induction). Each horizontal line shows the presence of XRCC1 (red), PCNA (green), or both (yellow) in a SSB site in each cell. The recruitment of XRCC1 and PCNA to the same damage site indicates active SP BER and LP BER. The activity of LP BER was undetectable in ES and low in MS and LS.

The imaging of PCNA recruitment to the induced SSB was more difficult than XRCC1 due to the relatively low number of recruited molecules and consequently the low intensity of eGFP-PCNA fluorescence accumulated at the damage site (Figure 3). Within 10 minutes of damage induction, detectable accumulation of PCNA at SSB damage sites occurred in only 1 out of 3 nonreplicating cells (Figure 4(a)).

In mid and late S phase, PCNA recruitment to damage was seen in even fewer cells (8% and 16%, respectively) (Figure 4(a)). No recruitment was detected in the early S phase. Unlike in the case of XRCC1, due to the low signal-to-noise ratio (and the presence of a mobile pool of eGFP-PCNA in live cells), the foci did not appear as distinct, sharply defined dots in the images of live or fixed cells (Figure 3(a,c), Suppl. Figure S5).

### Involvement of LP and SP BER in SSB repair in various stages of the cell cycle

PCNA is involved in LP but not in SP sub-pathway of BER [6,21,22]. We asked whether involvement of PCNA in replication limits its availability for SSB repair by LP BER. This limitation could be reflected in a smaller fraction of SSBs repaired by LP BER in the S phase compared to nonreplicating cells. To assess the activity of SP and LP BER in nonreplicating and replicating cells, we examined the recruitment of both PCNA and XRCC1 to the same sites of laser-induced SSBs in cells expressing both fluorescent fusion proteins. The position of an imaged cell in the cell cycle phase was determined on the basis of the pattern of PCNA distribution in the cell (Suppl. Figure S2). The substages that were distinguished are early S phase (ES), mid S phase (MS), and late S phase (LS) ([23,24]).

Both PCNA and XRCC1 were recruited to SSB damage sites in approximately 33% of nonreplicating cells. We also detected some recruitment of PCNA and XRCC1 to single-strand breaks in MS and LS but not in ES (Figure 4). In the early S phase only mRFP-XRCC1 was recruited and no accumulation of eGFP-PCNA was detected at the damage sites. These observations suggest that both the LP and SP subpathways are active in nonreplicating, MS, and LS phase cells, with SP BER repairing lesions in more than 60% of nonreplicating cells, and in even higher percentage of cells in MS and LS. The contribution of LP BER to overall SSB repair was minor (Figure 4). We associate the higher share of SSB repair by LP BER in nonreplicating cells in comparison with S phase cells with the larger number of PCNA molecules in an unengaged mobile pool. This abundant mobile pool of eGFP-PCNA is seen in images of nonreplicating cells (Figure 3(bc), Suppl. Figure S5). The activity of LP BER in MS and LS could also arise from the growing availability of unengaged PCNA, but we were unable to obtain reliable measurements of the mobile fraction of this protein in transfected cells due to variable levels of expression of the fusion protein.

### Saturation of the cellular response to SSBs

Cells have efficient SSB repair mechanisms, but it is obvious that there must be a limit to the number of lesions that can be repaired simultaneously. This raises the question as to how many SSBs can a cell attempt to repair and which repair pathways are involved, short-patch (SP) or long-patch (LP).

To establish the number of SSBs that saturate the DNA damage response, we first induced various numbers of SSBs in cells in subsequent stages of the cell cycle and imaged the formation and dismantling of XRCC1 and PCNA-containing repair foci. SSBs were induced by focusing a laser beam in one site within the nucleus generating 11 ± 3 SSBs, and quickly moving to the next site (see Materials and Methods). Thus, these groups of SSBs were induced in succession, that is, within a time much shorter than the minimum time required for SSB repair. Five to 30 sites within the nucleus were locally illuminated; therefore, the total number of SSBs induced in one nucleus ranged, on average, from 55 to 330.

**Figure 5.**
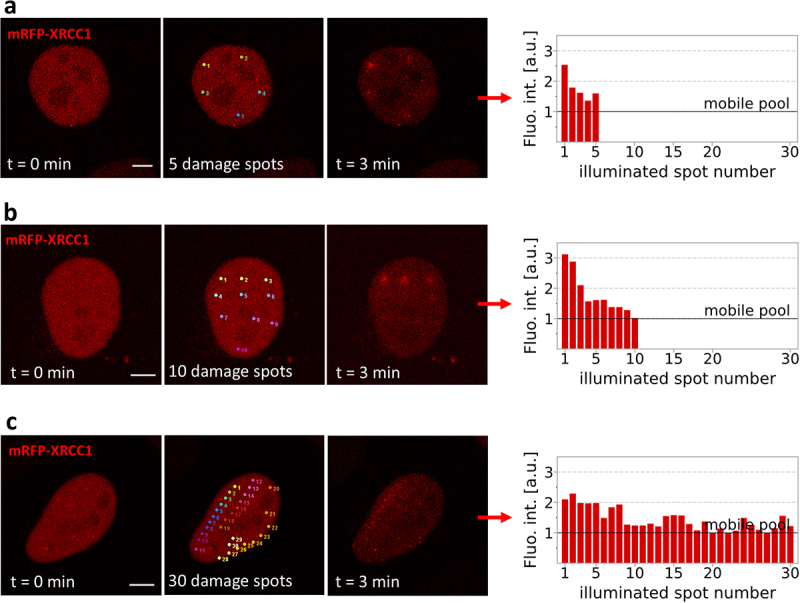
Response to various numbers of SSBs (55–330) induced in a cell nucleus of a cell expressing mRFP-XRCC1. Damage was induced in 5 (a), 10 (b), and 30 (c) sites (data for 15 and 20 sites are shown in Suppl. Figure S6). Images show mRFP-XRCC1 in the nucleus before damage induction (left), positions of damage sites (center), and foci of mRFP-XRCC1 recruited to damage sites (right). The graphs present fluorescence intensities of the accumulated mRFP-XRCC1 measured 3 min after damage induction, that is, at the time when the local concentration of the recruited protein reached a maximum. Scale bar 5 μm.

In cells where 5 nuclear sites were damaged (inducing approximately 55 SSBs in total), XRCC1 was recruited to all of them. The levels of fluorescence of mRFP-XRCC1 at the damage sites reached peak values 3–4 minutes after damage induction (Figure 2(b)). The intensity of fluorescence of the accumulated XRCC1 was the highest at the first damage site and was lower by a similar factor at each subsequent site (Figure 5(a)).

When SSBs were induced at 10–30 sites in a nucleus, the recruitment of XRCC1 usually occurred only at several (approximately 10) sites damaged first, with minimal or no detectable recruitment at other sites where damage was induced later (Figure 5(b,c), Suppl. Figure S6). This suggests that cells can only respond to damage when the total number of new SSB (i.e., in addition to endogenous DNA lesions) in the nucleus does not exceed approximately 110. When the number of SSBs is higher, recruitment of XRCC1 is not observed.

We wondered whether a stress associated with transfection and expression of a fusion protein could influence the cellular reaction to damage. Control experiments in nontransfected cells, fixed 5 minutes after damage and stained with anti-XRCC1 antibodies, yielded results nearly identical to live cells (Suppl. Figure S7). Recruitment was again strongest at the first site and weaker and eventually undetectable at later sites. We conclude that transient expression of XRCC1 did not alter its recruitment dynamics or its capacity to simultaneously repair multiple lesions.

In all experiments, the amount of XRCC1 recruited to each subsequent damage site was lower than at the previous site (Figure 5, Suppl. Figure S6). This demonstrates that despite suffering several new lesions, a cell always commits only a fraction of the available XRCC1 to damage. Some detectable amount of XRCC1 always remained in the form of a mobile pool.

The decreasing numbers of XRCC1 molecules recruited to subsequent damage sites follow a pattern that resembles exponential decay. This suggests that the number of molecules committed to each subsequent damage site is proportional to the currently available mobile pool of XRCC1.

As the number of induced SSBs increases beyond approximately 110 and the available pool of XRCC1 decreases, its recruitment becomes undetectable. However, there is always a fraction of XRCC1 molecules (in the mobile pool) that are not recruited to the damage. A possible reason for stopping the process of committing XRCC1 to multiple damage sites, despite the presence of some protein still available, could be a lack of a signal that induces damage recruitment. One may hypothesize that this could be the ADP-ribosylation of proteins at the damage sites. The formation of ADP-ribose polymers (PARylation) by PARP1 is very fast and depletes cellular NAD^+^. Induction of a very high number of DNA lesions could lead to a significant depletion of NAD^+^. This, in turn, could bring about a stop to further PARylation and, consequently, a stop to XRCC1 accumulation at DNA damage sites [3,25] when the number of SSBs induced within a very short time is high, as was the case in the experiments described in Figure 5(b,c), and Suppl. Figure S6).

Interestingly, the fact that only a fraction of available XRCC1 was recruited to each subsequent SSB damage site induced within one second indicates that the process of committing this fraction to a given repair site takes less than a second. In other words, if the process of committing XRCC1 molecules to damage were to be slow and last tens of seconds or more, the pool available for subsequent damage sites would be similar for all lesions induced within a few seconds. In this case, the amount of recruited XRCC1 to all damage sites would be similar. Our data demonstrate that the available pool of XRCC1 is significantly reduced after each new group of 11 SSBs is induced in one second. If extensive ADP-ribosylation and NAD^+^ depletion are indeed responsible for the halt of XRCC1 recruitment to numerous DNA lesions, it follows that extensive PARylation and NAD^+^ depletion occur within less than a few seconds after damage induction, which is even faster than the earlier estimate of several seconds to minutes [26–29].

We attempted to measure and compare the saturation of LP BER and SP BER by imaging not only XRCC1 but also PCNA recruitment to induced SSBs. A lack of PCNA recruitment could reveal a complete halt to LP BER in cells with numerous SSBs. However, due to the low intensity of the signals of PCNA recruited to damage, it was not possible to reliably detect differences between the amounts of accumulated protein (Suppl. Figure S8). This precluded establishing whether high levels of DNA damage resulted in damage-dependent inhibition of PCNA recruitment.

### Cellular response to two sets of SSBs induced minutes apart

**Figure 6.**
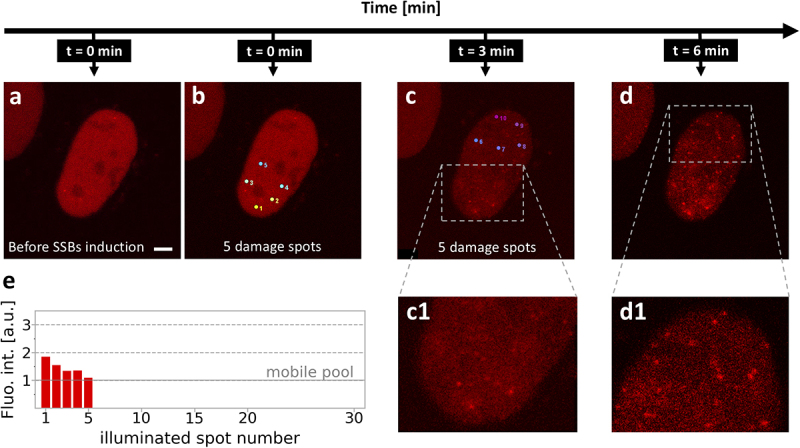
DNA damage response to induction of two sets of DNA damage, separated by a 3 min interval, showing the formation of subnuclear bodies. (a) Image of mRFP-XRCC1 before damage induction, (b) positions of damage sites, (c) image of mRFP-XRCC1 3 minutes after the first damage, with positions of the second set of damage sites marked (c1 – higher magnification), (d) image of mRFP-XRCC1 3 minutes after induction of the second set of lesions. XRCC1 is found in numerous subnuclear foci (d1 – higher magnification), (e) a graph showing the intensity of recruited XRCC1, measured at the damage sites 4 min after damage. Scale bar 5 μm.

When two sets of SSBs are induced a few minutes apart (Figure 6), the XRCC1 response surprisingly differs from the one observed in the case of an equal number of SSBs induced in close succession (that is, from the one presented in Figure 5(b,c)). We first induced SSBs in five closely located sites at the periphery of the nucleus (Figure 6b), which led to foci formation (Figure 6(c,c1)). 3 min later, additional SSBs were induced at five sites at a distant end of the nucleus (Figure 6c, the upper part of the nucleus). The latter SSBs did not result in accumulation of XRCC1 at the damage sites. Careful examination of the images showed that numerous subnuclear XRCC1-containing foci were formed at positions that were not related to the damage sites (Figure 6(d, d1)). This observation suggests that a fraction of XRCC1 that was not recruited to the first damage site, given a few minutes without additional damage, formed new subnuclear bodies (or entered existing ones). A possibly related phenomenon of forming numerous XRCC1-containg mobile subnuclear foci in nuclear regions away from the induced damage was reported previously ([5] – Suppl. movie 2). It is tempting to speculate that within a few minutes of damage induction, the mobile pool of XRCC1 is mobilized in preassembled repair complexes throughout the cell nucleus.

## Conclusions

The data presented above lead to the following conclusions.

- SP BER is active throughout the cell cycle and repairs SSBs in more than 60% of nonreplicating cells. LP BER is active in 33% of nonreplicating cells, becomes inactive in ES, and shows activity again in MS and LS (in 8 and 16% of cells, respectively).
- Cells retain a capacity to respond to damage if the total number of SSBs induced within seconds does not exceed approximately 110; Induction of more breaks does not evoke XRCC1 recruitment.
- Within less than a second, a cell commits a part of the currently available pool of XRCC1 molecules to accumulation at the damage site, thus the amount accumulated at subsequently induced SSBs gradually decreases.
- We hypothesize that a limit on the number of SSBs which elicit BER response arises from exhaustion of the signal required for recruitment; one possibility could be a limited capacity to produce poly-ADP-ribose in response to induction of a high number of SSBs.

## Supplementary Material

SZELEST_SUPPL_MAT_23_03_2025_JD2.docx

## Data Availability

Experimental data are available at: https://uj.rodbuk.pl/dataset.xhtml?persistentId=doi:10.57903/UJ/0CRDUY
