# Supplementary material for: Repair of single-strand breaks during the cell cycle - saturation of repair capacity
Source: Nucleus. 2026 May 17;17(1):2673717. doi: 10.1080/19491034.2026.2673717 (PMC13182976; doi:10.1080/19491034.2026.2673717)
Supplement: SZELEST_SUPPL_MAT_23_03_2025_JD2.docx [file KNCL_A_2673717_SM4814.docx]

**Supplementary Information**

**to**

**Repair of single-strand breaks during the cell cycle - saturation of repair capacity**

Oskar Szelest, Agnieszka Hoang-Bujnowicz, Jurek W.Dobrucki*, Mirosław Zarębski*

**Suppl. Fig. S1.** An image of DNA in the cell nucleus overlaid with the illumination spot formed by a focused laser beam that was used to induce local DNA damage. Illuminating a cell with a focused beam of visible light is used in this work as a precise tool for generating SSBs in a small region of the cell nucleus.

**Suppl. Fig. S2.** Typical patterns of eGFP-PCNA in the nuclei of cells that progress through the cell cycle.

**Suppl. Fig. S3.** An example of the recruitment of mRFP-XRCC1 to local DNA damage.

**Suppl. Fig. S4.** The percentage of cells in which the mRFP-XRCC1 recruited to damage was still detected within 1–10 min after induction of SSBs.

**Suppl. Fig. S5.** An example of the recruitment of eGFP-PCNA to locally induced DNA damage.

**Suppl. Fig. S6.** DNA damage response to induction of multiple SSBs at 15 or 20 damage sites within a cell nucleus detected by recruitment of mRFP-XRCC1.

**Suppl. Fig. S7.** Recruitment of XRCC1 to DNA damage induced at 15 damage sites within a cell nucleus, detected by immunofluorescence.

**Suppl. Fig. S8.** Recruitment of eGFP-PCNA to DNA damage induced at 10 sites within a cell nucleus.


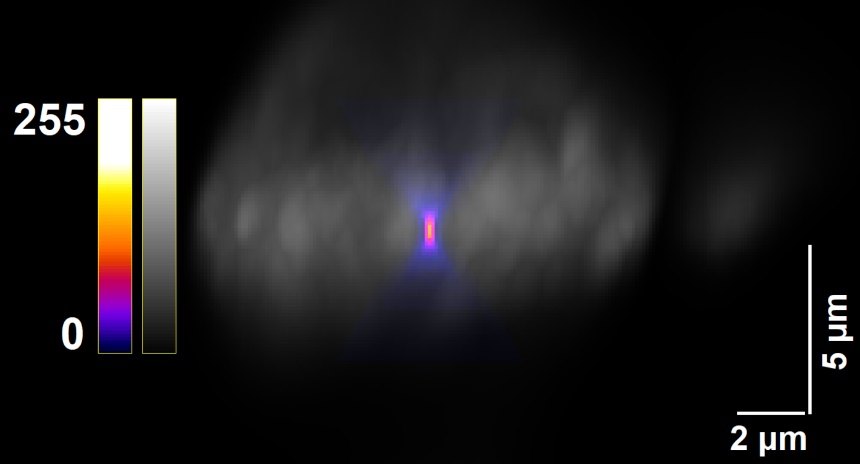


**Supple. Fig. S1.** An image of DNA in the cell nucleus overlaid with the illumination spot formed by a focused laser beam that was used to induce local DNA damage. The fluorescence maximum intensity projection image of a HeLa cell stained with DAPI (pseudocolored in grayscale) was combined with a projection of the simulated intensities of the illumination focus (pseudocolored using the Fire LUT) produced with the PSF Generator plugin for Fiji (ImageJ) [1]. The region of the highest irradiance is confined to a small central volume marked with a yellowish ellipsoid. The laser light intensity and DAPI fluorescence color and gray scales are shown on the left.

Here illuminating a cell with a focused beam of 488 nm light serves a precise tool for generating SSBs in a small region of the cell nucleus, as described below.

It is possible to precisely induce DNA damage in a defined position within a cell on a microscope stage by focusing laser light into a diffraction-limited spot. The spatial probability of inducing damage is proportional to the irradiance of the illumination light, which in the Born and Wolf model [2] is a 3-dimensional function with cylindrical symmetry around the optical axis. According to this model, over 80% of the energy is delivered to a central region measuring approximately 320 nm in diameter in the imaging plane when the blue light is focused with a high numerical aperture objective lens. The approximate average photon flux in this region is highest in the imaging plane and decreases six times at a distance of 600 nm along optical axis (Suppl. Fig. S8). This means that the probability of inducing damage in this region is reduced 6 times. The calculations were based on the 3D image stacks generated in PSF Generator Plugin for ImageJ [1].

By increasing the total energy delivered by the focused laser light, the expected number of induced DNA breaks is expected to be higher. However, each individual break has the same spatial probability of occurrence, proportional to local illumination intensity. The expected number of SSBs induced in our experiments by delivering 120 μJ of 488 nm CW laser light (with an intensity of 120 μW) focused into a diffraction-limited spot was estimated to be 
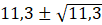
 (based on a reasonable assumption that photon-induced DNA damage adheres to Poisson statistics). Therefore, such a low number of total breaks means that they are effectively induced in the region of the highest irradiance, that is, in the center of the illumination focus, while the probability of inflicting damage in the surrounding regions is very low.

**
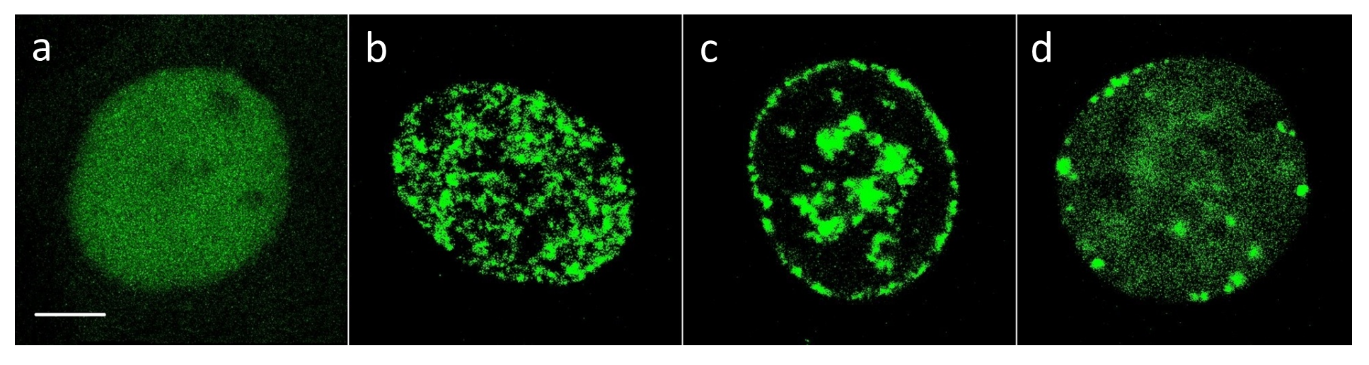
**

**Suppl. Fig. S2.** Typical patterns of eGFP-PCNA in the nuclei of cells that progress through the cell cycle.

1. In nonreplicating cells, eGFP-PCNA exists in a form of a mobile pool distributed evenly throughout the cell nucleus.

**b-d.** During DNA replication eGFP-PCNA forms patterns [3,4] characteristic for early **(b)**, mid **(c),** and late **(d)** S phase. Scale bar 5 µm.

**
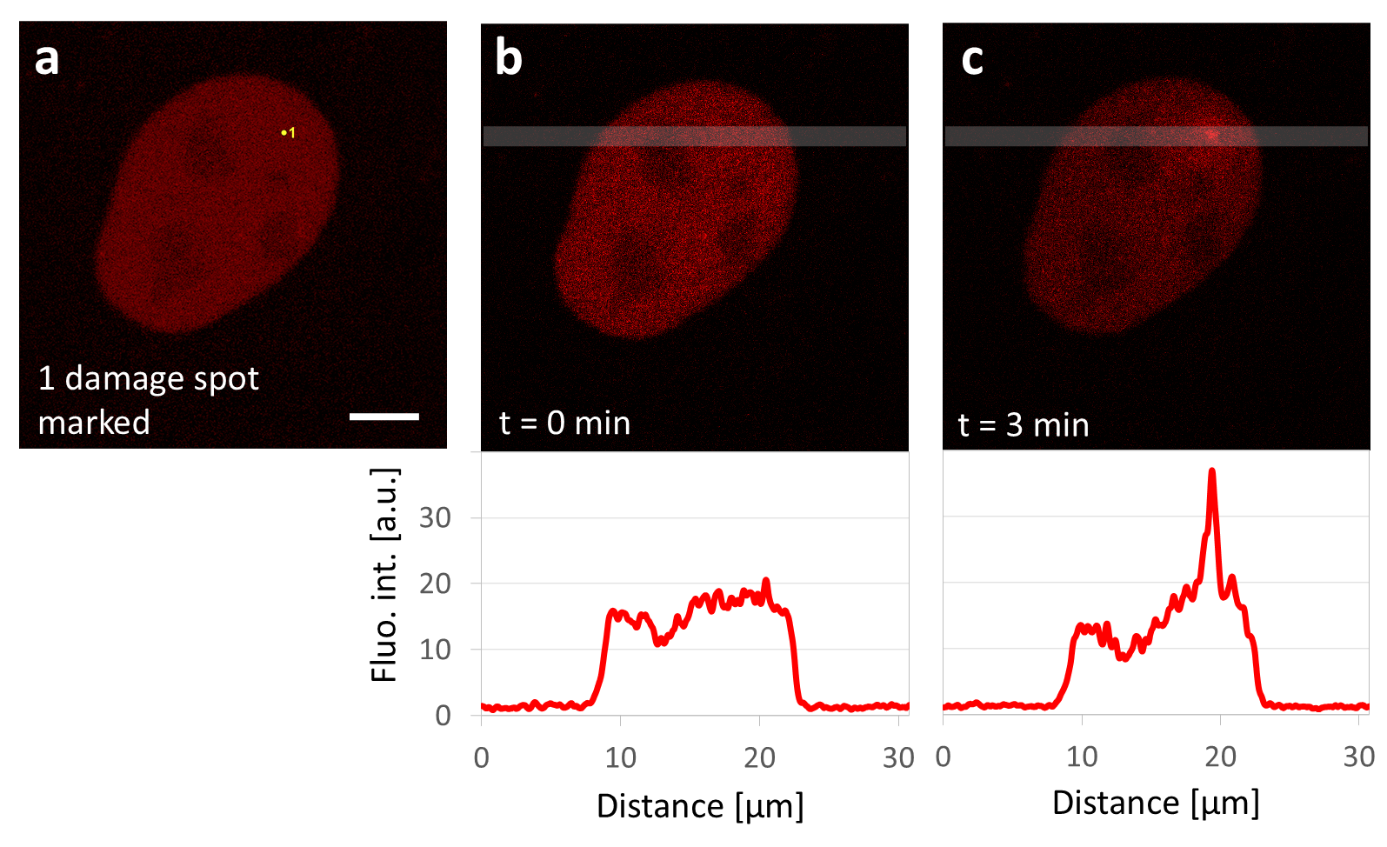
**

**Suppl. Fig. S3.** An example of the recruitment of mRFP-XRCC1 to local DNA damage.

Representative images and corresponding fluorescence intensity profiles in a cell expressing mRFP-XRCC1. Inducing 11±3 SSBs with a beam of blue light focused on a single selected spot in the cell nucleus (indicated in **(a)**) results in a rapid recruitment of mRFP-XRCC1 (shown in (c)). Fluorescence intensity profiles (plotted along a transparent gray line that runs across the damage site) represent relative local concentrations of mRFP-XRCC1 before damage induction **(b)** and 3 min after damage induction **(c)**. 3 min after damage, the fluorescence intensity of the protein recruited to damage is approximately 2.5 times higher than that of a mobile pool in the surrounding nucleoplasm. Scale bar 5 µm.

**
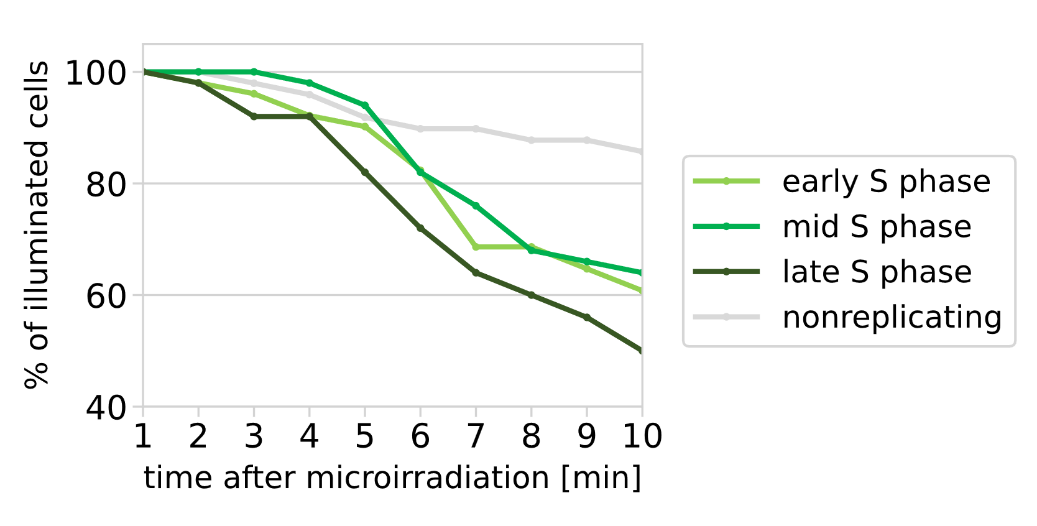
**

**Suppl. Fig. S4.** The percentage of cells in which the mRFP-XRCC1 recruited to damage was still detected within 1–10 min after induction of SSBs in a 2.5 µm × 2.5 µm region.

**
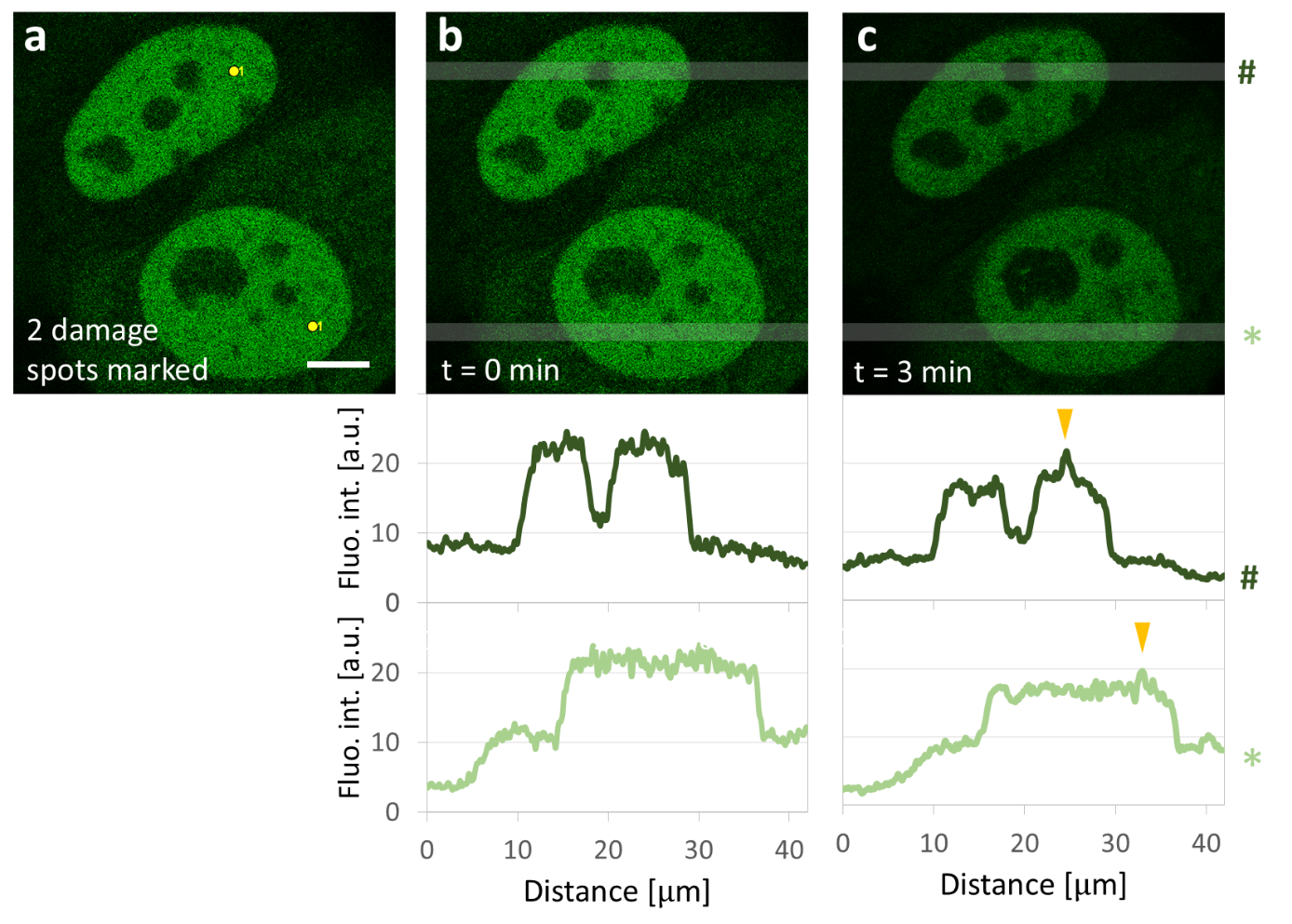
**

**Suppl. Fig. S5.** An example of the recruitment of eGFP-PCNA to locally induced DNA damage.

Representative images and corresponding fluorescence intensity profiles are shown for nonreplicating cell nuclei expressing eGFP-PCNA. Inducing 11±3 SSBs with a beam of blue light focused on a single selected spot in the cell nucleus (marked 1 in **(a)**) results in the recruitment of eGFP-PCNA (shown in (c)). Fluorescence intensity profiles along the transparent gray lines (indicated by **(#)** or **(*)**) represent relative local concentrations of eGFP-PCNA before **(b)**, and 3 min after damage induction **(c)**. The accumulation of eGFP-PCNA is detected in the fluorescence intensity profiles, as highlighted by the orange arrows that mark the locations of the damage. Scale bar 5 µm.

**
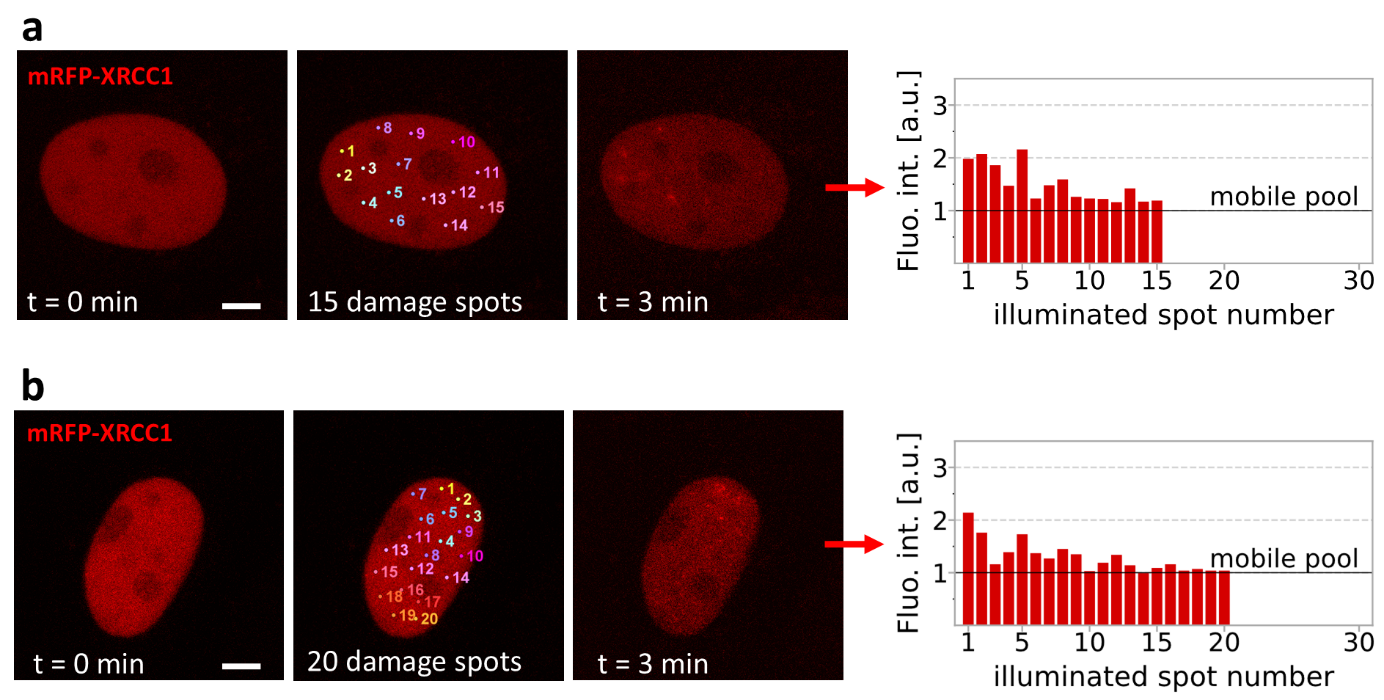
**

**Suppl. Fig. S6.** DNA damage response to induction of multiple SSBs at 15 or 20 damage sites within a cell nucleus detected by recruitment of mRFP-XRCC1.

DNA damage was induced in the nuclei of cells expressing mRFP-XRCC1 by focusing blue laser light on 15 **(a)** or 20 sites **(b)** within the cell nucleus. Images represent distribution of mRFP-XRCC1 in the cell nucleus before damage induction (left), the positions of the induced damage (center), and foci of mRFP-XRCC1 recruited to the damage sites (right). The graphs represent the fluorescence intensities of mRFP-XRCC1 accumulated at each damage site, determined 3 min after damage induction. Scale bars 5 µm.


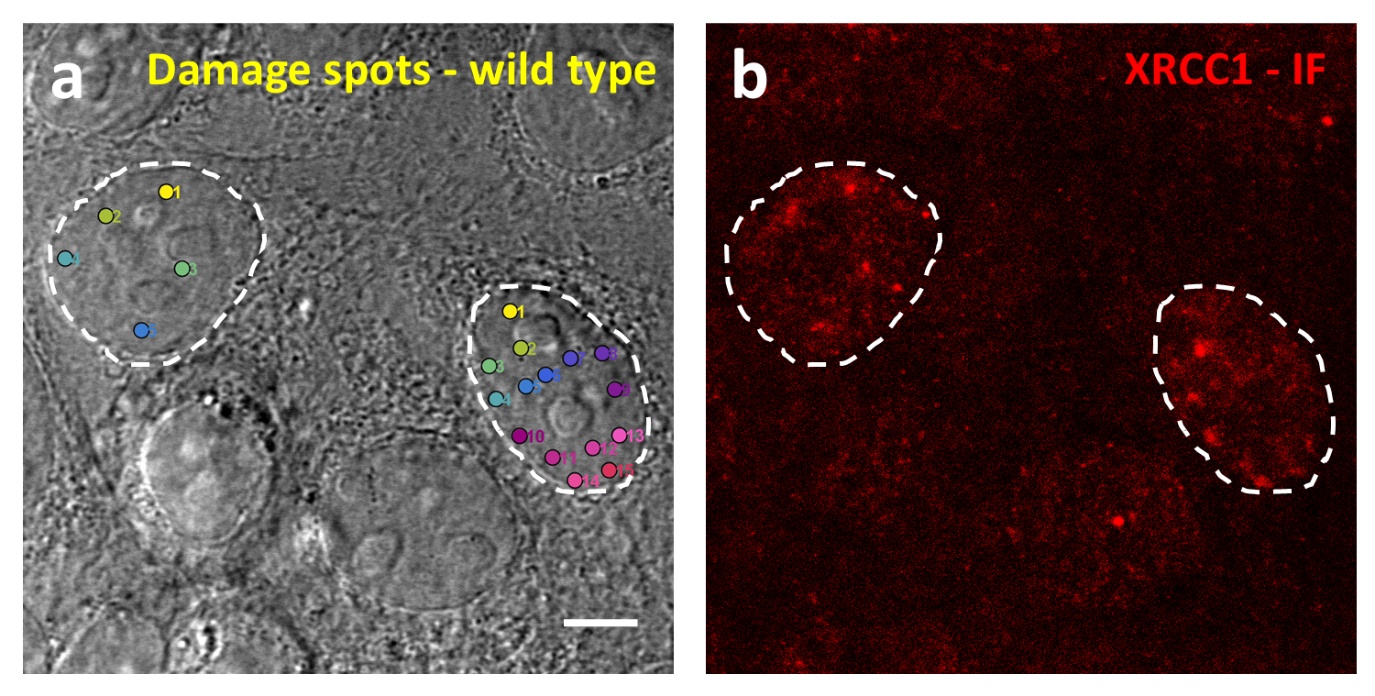


**Suppl. Fig. S7.** Recruitment of XRCC1 to DNA damage induced at 15 damage sites within a cell nucleus, detected by immunofluorescence.

DNA damage was induced by a beam of focused blue light in the nuclei of wild-type HeLa cells. XRCC1 was detected by immunofluorescence (IF) 3 minutes after damage induction. The images show the positions of the damage sites (**a**) and foci of XRCC1 that was recruited to the damage sites (**b)**. Scale bar 5µm.

**
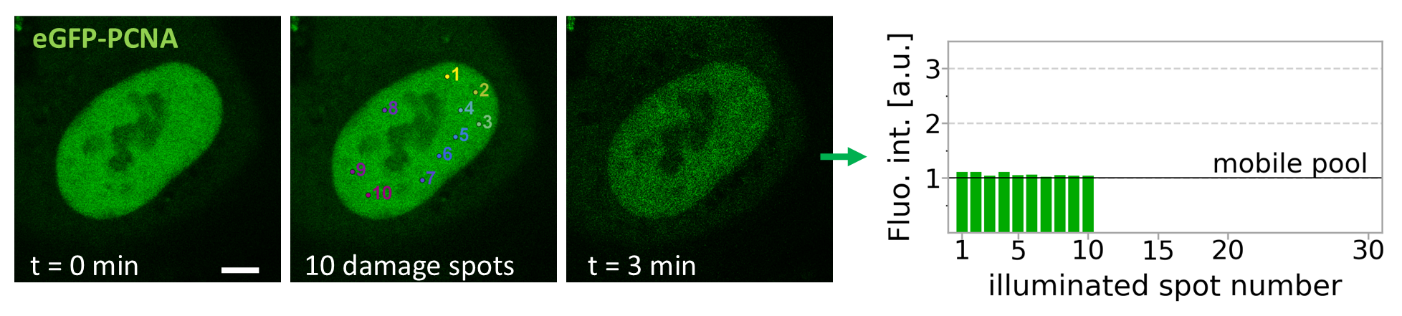
**

**Suppl. Fig. S8.** Recruitment of eGFP-PCNA to DNA damage induced at 10 sites within a cell nucleus.

DNA damage was induced in the nuclei of cells expressing eGFP-PCNA by focusing the blue laser light at 10 distinct sites. Images show the distribution of eGFP-PCNA in the cell nucleus before damage induction (left), positions of the damage sites (center), and foci of eGFP-PCNA recruited to the damage sites (right). The graph presents the fluorescence intensities of the accumulated eGFP-PCNA measured 3 min after damage induction. Scale bar 5 µm.

**References**

1. Kirshner H, Aguet F, Sage D, Unser M. 3-D PSF fitting for fluorescence microscopy: implementation and localization application. J Microsc. 2013;249:13–25. https://doi.org/10.1111/j.1365-2818.2012.03675.x

2. Born M, Wolf E, Bhatia AB, Clemmow PC, Gabor D, Stokes AR, et al. Principles of Optics. Cambridge University Press; 1999. https://doi.org/10.1017/CBO9781139644181

3. Leonhardt H, Rahn HP, Weinzierl P, Sporbert A, Cremer T, Zink D, et al. Dynamics of DNA replication factories in living cells. J Cell Biol. 2000;149:271–80. https://doi.org/10.1083/jcb.149.2.271

4. Somanathan S, Suchyna TM, Siegel AJ, Berezney R. Targeting of PCNA to sites of DNA replication in the mammalian cell nucleus. J Cell Biochem. 2001;81:56–67. https://doi.org/10.1002/1097-4644(20010401)81:1<56::aid-jcb1023>3.0.co;2-#
